# Do Items Addressing Thoughts and Emotions Regarding Symptoms Measure Distinct Aspects of Musculoskeletal Health?

**DOI:** 10.1177/23743735231211776

**Published:** 2023-11-06

**Authors:** Sina Ramtin, Lee Reichel, David Ring, Sean Gallagher, Teun Teunis

**Affiliations:** 1 Department of Surgery and Perioperative Care, 377659 Dell Medical School, The University of Texas at Austin, Austin, Texas, USA; 2 Department of Plastic Surgery, University Pittsburgh Medical Center, University of Pittsburgh, Pittsburgh, Pennsylvania, USA

**Keywords:** emotions regarding symptoms, thoughts regarding symptoms, musculoskeletal, item groupings

## Abstract

A prior experiment identified separate thought and feeling item groupings among items in measures of unhelpful thinking (ie, catastrophic thinking, kinesiophobia). This study sought to confirm the utility of separating these factors using a subset of selected items. One hundred and thirty-six adult patients visiting a musculoskeletal specialist completed the surveys. Confirmatory factor analysis measured the association between variation in scores on a specific item with variation in scores in separate groupings for thoughts and feelings, and a combined item grouping. Cronbach alpha (internal consistency) and Spearman correlation with magnitude of capability were also measured for the three separate item groupings. The association of variation in specific items with variation in a group of items addressing thoughts, a group of items addressing feelings, and the combination of all items was comparable. The internal consistency and strength of association with magnitude of capability were also comparable. The finding of no advantage to separation of items addressing thoughts and feelings regarding symptoms suggests that just a few items may be able to represent unhealthy mindsets regarding musculoskeletal symptoms.

## Introduction

### Background

Unhelpful thoughts and feelings of worry or despair (distress) regarding symptoms account for a notable proportion of the variation in magnitude of incapability among individuals seeking musculoskeletal specialty care.^1-10^ For instance, people report more intense pain and greater incapability when painful movement is interpreted as making their condition worse, more so when they are also feeling symptoms of worry or despair.^11^ The current measures of unhealthy mindsets regarding symptoms use varied characterizations that seem to mix items that address unhelpful thoughts (cognitive errors or misconceptions) with items that address unhelpful feelings such as worry and despair regarding symptoms.^12-14^ In a prior study, we performed factor analysis using the items from the Pain Catastrophizing Scale (PCS), Negative Pain Thoughts Questionnaire (NPTQ), Intolerance of Uncertainty Scale (IUS), and Tampa Scale of Kinesiophobia (TSK) and identified two distinct statistical groupings of items (factors), one of which was clearly related to unhelpful thoughts (things that are true or false) and the other related to unhelpful feelings (feelings of distress [worry or despair], which are personal and not verifiable), each contributing independently to variation in magnitude of incapability.^15^ On the other hand, the two factors were highly correlated and more experiments are needed to determine if there are statistically distinct factors addressing thoughts and feelings.

### Rationale

There is some evidence in psychological experiments that it is not possible to clearly distinguish the effects of thoughts and feelings.^16^ And yet, initial work on statistical groupings of distinct levels of these factors do associate with independently with level of capability.^17,18^ Furthermore, thoughts and feelings might raise different points of discussion or lead to specific approaches to comprehensive, mental, and physical health treatment strategies that could make them more appealing for individuals. For instance, given that distress makes unhelpful thoughts more difficult to reorient,^11^ it might be important to address worry and hopelessness prior to addressing unhelpful thinking. In the realm of musculoskeletal symptoms, more study is needed to determine an efficient set of items that capture the most relevant aspects of mental health, specifically whether it is worthwhile to measure and address thoughts and feelings separately. Our objective was to further advance and authenticate the approach of inquiring about distress and thoughts regarding symptoms by posing a limited number of questions. The aim of the current study is to determine whether or not we can reproduce the separation between unhelpful thoughts and feelings about symptoms in a group of people with various orthopedic conditions and durations of symptoms using a more selective set of representative items.

### Study Questions

We collected a new set of questionnaires addressing unhelpful thoughts and feelings of distress regarding symptoms from a cohort patients seeking musculoskeletal specialty care, used exploratory factor analysis to identify separate thoughts and feelings groupings of items in the measures, and used these factors to address: (1) To what degree do items felt to measure unhelpful thoughts and unhelpful feelings represent their corresponding item grouping in confirmatory factor analysis (a test of how well specific items represent their group)? (2) Is there a difference in internal consistency of separate thought and feeling factors measured using Cronbach alpha (a measure of how the items in a group relate to one another) compared to a single, combined set of items addressing both thoughts and feelings? (3) Is there a difference in the association of separate thought and feeling factors with magnitude of capability compared to a single, combined set of items addressing both thoughts and feelings?

## Methods

### Study Design and Participants

We enrolled 136 new and return adult, English-speaking patients in a cross-sectional study of people seeking musculoskeletal specialty care of upper extremity, lower extremity, or spine conditions in one of four offices in a large urban area in US from August 2021 to October 2021. We purposely included both new and return patients in the current phase of the project for a representative spectrum of patients presenting for musculoskeletal specialty care. The study was approved by our institutional review board. Ten patients (7.3%) did not complete all questions, perhaps because of a technical problem when completing the survey. Some people may have done the survey on their phone using a QR code when the unit's research tablet was occupied by another patient. We know that people are less likely to complete surveys on their own phone than on our tablet. Factor analysis can only analyze complete cases. Multiple imputation, the ideal method of addressing missing data at random, cannot be used for factor analysis. We therefore omitted all 10 patients who did not complete all questionnaire, leaving 126 patients for analysis.

### Selection of Mental Health Items

Prior work suggests that commonly used mental health questionnaires combine different aspects of mental health. We identified items measuring unhelpful thoughts and unhelpful feelings as distinct entities associated with magnitude of incapability.^13^ We determined which items of the validated Pain Catastrophizing Questionnaire (PCS-13), Tampa Scale of Kinesiophobia (TSK-11), and Negative Pain Thoughts Questionnaire (NPTQ-11) measure unhelpful thoughts and unhelpful feelings about symptoms. We selected specific items from the PCS-13, TSK-11, and NPTQ-11 for this study according to the following criteria: (1) Good prior performance in both exploratory and confirmatory factor analysis;^13^ (2) Items that address verifiable facts to represent the “thoughts” factor and items that address personal feelings for the “feelings” factor. We limited the number of items because we are aiming for a brief measure and based on the analysis, all of the items are comparably representative. Unhelpful thoughts are statements that are not true such as “Pain always means I have injured my body” or not true for the conditions under consideration such as “My body is telling me I have something seriously wrong.” Such items represent common misinterpretations of symptoms that are associated with greater pain intensity and magnitude of incapability. The items used to measure unhelpful thoughts included items 3, 5, 6, and 10 from the TSK-11 and item 10 from the NPTQ-11 (Table 1). We used the term “feeling” to describe emotions that are subjective and not objectively verifiable. This is distinct from thoughts that can be objectively demonstrated as correct or incorrect, such as “I am incapable of performing activities that regular individuals can do because I am prone to injury.”

**Table 1.** Proposed Question Groupings.

Question groupings	Item	Question
Unhelpful feelings	PCS 1	I worry all the time about whether the pain will end.
	PCS 5	I feel I can't stand it anymore.
	PCS 10	I keep thinking about how much it hurts.
	PCS 11	I keep thinking about how badly I want the pain to stop.
	NPTQ 1	My problem makes me feel awful and it overwhelms me.
	NPTQ 4	I should not feel this way, I should not have this problem.
Unhelpful thoughts	NPTQ 10	I will never be happy again as long as I have pain.
	TSK 3	My body is telling my I have something dangerously wrong.
	TSK 5	My accident/problem has put my body at risk for the rest of my life.
	TSK 6	Pain always means I have injured my body.
	TSK 10	I can't do all the things normal people do because it's too easy for me to get injured.

Abbreviations: PCS, Pain Catastrophizing Scale; NPTQ, Negative Pain Thoughts Questionnaire; TSK, Tampa Scale of Kinesiophobia.

To represent unhelpful feelings we used items 1, 5, 10, and 11 from the PCS-13 and Items 1 and 4 from the NPTQ-11. These items are personal rather than factual and they represent feeling worried, overwhelmed, despairing, and desperate—personal factors that are not true or false. Examples include feeling overwhelmed “I feel I can't stand it anymore” and rumination “I keep thinking about how badly I want the pain to stop” (Table 1).

Responses on all the thoughts and feelings items were measured on a 5-point Likert scale: 1 (“not at all”) to 5 (“all the time”).

### Capability Questionnaires

The PROMIS physical function (PF) computer adaptive testing (CAT) assesses the magnitude of physical capability. A score of 50 represents the mean and every 10 points away from the mean represent a standard deviation away from the mean for a general population in the US Higher scores reflect greater capability.

### Other Variables

Patients also completed a survey of age, sex, marital status, education level, household income, insurance type, anatomic region, traumatic diagnosis (yes/no), visit type (new/return), and specific clinician (Table 2).

**Table 2.** Patient Demographics.

Variable	Value
No. of patients	126
Age (yr)^a^	50 ± 18
Gender	
Women	52% (66)
Men	47% (59)
Other	0.79 (1)
Marital status	
Married or partner	50% (63)
Single	36% (45)
Divorced or separated	14% (18)
Education	
High school or lower	21% (27)
2-year college	16% (20)
4-year college	32% (40)
Postgraduate degree	31% (39)
Employment status	
Employed	61% (77)
Retired	22% (28)
Unemployed	9.5% (12)
Other	7.1% (9)
Annual household income	
Less than $24,000	14% (18)
Between $24,000 and $45,999	14% (18)
Between $46,000 and $74,999	17% (21)
Between $75,000 and $121,000	18% (23)
More than $121,000	37% (46)
Insurance type	
Commercial	60% (76)
Medicare	21% (27)
Other	18% (23)
Anatomic region	
Upper extremity	67% (84)
Hip or knee	25% (32)
Other	7.9% (10)
Traumatic diagnosis	
No	57% (72)
Yes	43% (54)
Visit type	
New	53% (68)
Return	47% (59)
Provider	
1	18% (23)
2	27% (34)
3	13% (16)
4	7.9% (10)
5	9.5% (12)
Other	25% (31)
PROMIS physical function (t-score)^a^	46 ± 10

Abbreviation: PROMIS, Patient-Reported Outcomes Measurement Information System.

^a^ Continuous variables presented as mean ± standard deviation; discrete variables as the percentage of patients, with the number in parentheses.

### Descriptive Data

The patients were 53% women (71 of 126). The mean age of participants was 50 years (SD 18). We included 84 (67%) people with upper extremities conditions, 32 (25%) with lower extremities conditions, and 10 (7.9%) with other musculoskeletal conditions (Table 1).

### Primary and Secondary Study Outcome

Our primary goal was to assess if we can reproduce the separation between unhelpful thoughts and feelings about symptoms using a more selective set of items. We therefore compared the concept of two groups of items (thoughts vs feelings) to the concept of a single group of items (thoughts and feeling combined).

First, we used confirmatory factor analysis to measure how much variation in scores on a specific item account for one unit variation in the underlying item grouping (factor). Confirmatory factor analysis (CFA) is a measure of how well specific items represent the set of items they grouped with (the factor) in exploratory factor analysis. If there are two distinct thought and feeling item groupings, then separating thoughts and feelings gives a better representation of the data. The confirmatory factor analysis using two item groupings will explain more the variation within the data than a single group of items that does not make the distinction between thoughts and feelings. If there are two distinct factors (one measuring thoughts and the other measuring feelings) and our analysis falsely identified a single factor, the individual items would explain less of the variation in the inappropriately combined factor than we observe. If there is only one factor, the individual items would be expected to explain a similar amount of variation of the distinct factor and the combined factors, because both are essentially the same, and that is what we observed.

Second, we compared the internal consistency of two item groupings versus one combined using Cronbach alpha, which assigns a value to the degree to which each item is related to the other items in the grouping.

Third, we determined the correlation of each item grouping with magnitude of capability (PROMIS PF CAT) using Spearman rank correlation. We created 95% confidence intervals through bootstrapping (n = 1000). If the 95% confidence intervals overlap, there is no difference in the association with capability.

### Power Analysis

We powered on our third study question, because the power analysis is more straightforward. An a priori sample size calculation indicated that 124 participants would provide 80% power, with alpha set at 0.05, to detect a correlation of 0.25. We included 5% more people to account for missing data. In addition, previous simulation study indicates that our study should obtain excellent reliability (assuming a reliability criterion 0.98, a variable-to-factor ratio of 6:1, and a 2-factor solution) with 55 participants assuming high communality (factor loading between 0.6 and 0.8), and 95 assuming a wide communality (factor loading between 0.2 and 0.8).^19^

## Results

### How Well Do Thoughts and Feelings Items Represent Their Group?

There was no difference in the degree to which each item represented the overall item grouping when we compared groups of items that address thoughts and feelings about symptoms separately, compared to a set of items representing a combination of thoughts and feelings (Table 3).

**Table 3.** Comparing 1 and 2 Groups of Questions.

Question groupings	2 groups of questions					1 group of questions				
Unhelpful feelings	Coefficient (95% CI)	Standard error	Variation explained	Cronbach alpha	Correlation with PROMIS PF*	Coefficient (95% CI)	Standard error	Variation explained	Cronbach alpha	Correlation with PROMIS PF*
PCS 1	0.79 (0.72 to 0.87)	0.036	0.63	0.91	−0.45 (−0.60 to −0.29)	0.79 (0.72 to 0.86)	0.036	0.63	0.93	−0.50 (−0.64 to −0.36)
PCS 5	0.80 (0.73 to 0.87)	0.035	0.65			0.80 (0.73 to 0.87)	0.035	0.64		
PCS 10	0.78 (0.70 to 0.86)	0.039	0.61			0.78 (0.70 to 0.85)	0.039	0.61		
PCS 11	0.81 (0.74 to 0.88)	0.035	0.66			0.81 (0.74 to 0.88)	0.034	0.66		
NPTQ 1	0.87 (0.81 to 0.92)	0.027	0.75			0.87 (0.82 to 0.92)	0.026	0.75		
NPTQ 4	0.70 (0.60 to 0.79)	0.049	0.49			0.70 (0.60 to 0.79)	0.049	0.49		
Unhelpful thoughts										
NPTQ 10	0.79 (0.71 to 0.86)	0.038	0.62	0.83	−0.51 (−0.65 to −0.36)	0.79 (0.71 to 0.86)	0.038	0.62		
TSK 3	0.78 (0.70 to 0.86)	0.039	0.61			0.78 (0.70 to 0.85)	0.038	0.61		
TSK 5	0.68 (0.57 to 0.79)	0.054	0.46			0.68 (0.58 to 0.78)	0.051	0.46		
TSK 6	0.55 (0.42 to 0.67)	0.066	0.30			0.54 (0.42 to 0.67)	0.066	0.30		
TSK 10	0.70 (0.60 to 0.80)	0.051	0.48			0.69 (0.60 to 0.79)	0.049	0.48		

Abbreviations: CFA, Confirmatory factor analysis; PCS, Pain Catastrophizing Scale; NPTQ, Negative Pain Thoughts Questionnaire; TSK, Tampa Scale of Kinesiophobia.

*P value <0.05.

### Cronbach Alpha—Internal Consistency

Cronbach alpha was comparable for a single group of items (0.93) and for distinct thought and feeling groups of items (feelings about symptoms = 0.91; thoughts about symptoms = 0.83).

### Association With Capability

Since the correlation range of a single group of items and capability has more than 90% overlap with correlation range of two distinct groups of items separating thoughts and feelings and capability, there was no difference in the strength of association (Table 3).

## Discussion

Timely and accurate diagnosis of unhelpful thoughts and feelings of distress regarding symptoms has the potential to improve how people with musculoskeletal symptoms get and stay healthy. Motivated by a recognition that common measures of unhelpful thinking such as the PCS measure both feelings (distress/rumination) and unhelpful thinking (cognitive errors), we performed a factor analysis of items from measures we and others have tended to use to measure unhelpful thinking and identified separate constructs of unhelpful thoughts and feelings of distress. Specifically, we tested whether unhelpful thoughts about symptoms and feelings of distress about symptoms form separate statistical constructs (groupings) in a group of people presenting care for various musculoskeletal symptoms. We found no advantages to separate groupings of thought and feeling items, suggesting additional study is needed to determine if this distinction is worthwhile for specific pathologies or specific timepoints during illness.

This study has several limitations. First, the small number of incomplete responses was typical of some early pandemic enrollment methods. It is a small percentage of the sample and is unlikely to affect the results. Second, the generalizability of our findings might be limited by the fact that the majority of our patients were employed and had upper extremity conditions. In prior studies, cognitive factors were similar across socioeconomic factors and anatomical regions, so we do not anticipate much influence.^20,21^ If separate thought and feeling item groupings are established, they can be verified in varied samples and diverse populations. Third, we selected a subset of items we felt were representative of the separate thoughts and feelings constructs based in part on their performance scores in the prior factor analysis and in part on whether the items address facts or feelings. It is possible that this method of choosing items removed important information, or that factor analysis is not as reliable or accurate when a small number of items is used. Future experiments can test various combinations of constructs in varied settings. Fourth, factor analysis may be more robust with a larger number of participants. Studies in larger samples might have more consistent results. Fifth, the mixture of diagnoses, durations of symptoms, and duration in care might be important given the prior evidence found that the number of constructs related to mental health changes during recovery.^15^ Future studies can address the existence of separate thought and feeling constructs in specific settings such as within 2 weeks of musculoskeletal trauma. Sixth, we did not reproduce the two constructs observed in the prior study, as we did not utilize all of the items from the questionnaire employed in the prior study.^13^

### How Well Do Thoughts and Feelings Items Represent Their Group?

The observation that items addressing thoughts and items addressing feelings did not represent groupings of similar items better than they represented a combined thought/feeling grouping brings into question the potential utility of measuring thoughts and feelings separately. In the prior study that found 2 key constructs among items in the PCS, NPTQ, IUS measure, and TSK, one of which was related to unhelpful thoughts and the other relating more to unhelpful feelings of distress, the two identified constructs were highly correlated (r = 0.72, P < 0.001).^13^

There is a debate in psychology regarding whether it is useful or scientifically valid to measure thoughts and feelings separately.^22^ There is mounting evidence that emotions are socially constructed thoughts in response to physiological symptoms.^23^ Another line of research that links thoughts and emotions addresses rumination. Rumination, defined by the American Psychological Association as “excessive, repetitive thoughts or themes that interfere with other forms of mental activity,”^24^ is felt by some to be a foundation of symptoms of anxiety and depression. The idea is that iterative negative thinking interferes with problem-solving.^25-27^ Greater tendency to ruminate is associated with more negative emotions.^28^

Over the years, we have found that measures that address thoughts and feelings about symptoms (such as measures of catastrophic thinking, kinesiophobia, and negative pain thoughts) tend to correlate somewhat more strongly with symptom intensity and magnitude of capability than do general symptoms of worry or despair. This may be, in part, due to the observation that people seeking musculoskeletal care do not always complete measures of depression or anxiety forthrightly.^29-31^ Another important consideration is that there is some evidence that the number of constructs formed by mental health questionnaires is greater during the early stages of recovery from injury.^15^ Perhaps thoughts and emotions have important differences early on after an unexpected injury and when symptoms are most intense and the future less certain.

A final issue is that the strong interrelationships of the various mental health measures create the potential for unexpected results, perhaps as a result of collinearity. It may be helpful to represent mental health with a limited number of questions that feel relevant and important to patients and both quantify and bring to light important foci of treatment. Some people may be more interested in addressing thoughts than emotions.

### Cronbach Alpha—Internal Consistency

The observation that interquestionnaire correlation were notable for items addressing thoughts and items addressing feelings and combined thought/feeling grouping suggests that both concepts have good coverage and reliability which are important markers of validity, but that there may be little statistical advantage in separating them. Our findings are consistent with prior studies found that the items in the PCS, TSK-4, and NTPQ have adequate internal consistency.^32-35^ Our reproduction of the results regarding internal consistency also suggests that items addressing thoughts and items addressing feelings are notably related and may represent a singular mental health concept. Fusion with a thought about a symptom and distress about a symptom may be parts of a singular psychological process that is cumulative more so than synergistic as some mediation/moderation analyses have suggested.^11,36,37^ It may also be that there are synergistic effects, but they are non-linear as suggested in one study.^37^

### Association With Capability

The finding that there was no difference in the strength of association with capability between a single group of items compared with two distinct groups of items separating thoughts and feelings points to a potential benefit of discussing mental care opportunities from either perspective. There may or may not be a statistical advantage to separation of thoughts and feelings regarding symptoms, but there may be a therapeutic advantage if people are more comfortable addressing thoughts or feelings to start.^38^ The best approach may be to listen to patient concerns and follow their lead to open up discussions of how thoughts or feelings or both relate to pain intensity and magnitude of incapability,^38^ and how reorienting thoughts and alleviating distress can improve health.^39^ According to prior evidence, there are different ways of registering psychological health opportunities. Reflecting a patient's mindset on the face of clinicians endorses that the clinicians are able to detect the psychological aspects of conditions, whether these recognitions are implicit or explicit.^40^ Protective hand posturing and choosing words, and phrases can demonstrate a patient's mental health status and well-being that offer an important opportunity for more behavioral intervention.^41,42^

## Conclusions

The observation that items addressing thoughts and feelings separately did not outperform a combined thought/feeling grouping brings into question the potential utility of measuring thoughts and feelings separately. In the context of evidence from other studies that the number of constructs represented by these types of items reduces as recovery from an upper extremity fracture progresses,^15^ combined with the inconsistent results between two-factor analyses we performed, future studies might address specific settings such as early recovery after injury or surgery, perhaps separating arm and leg conditions. The concept of rumination—deep considered thought—might be relevant as it is often related to emotions such as despair or worry and could be the unifying construct that makes these seemingly different aspects of mental health so highly related. Perhaps the selection of a select few items to use might ultimately hinge on other factors such as the degree to which the item addresses something that matters to the patient and therefore seems relevant. Or perhaps, the degree to which a fruitful conversation is sparked that contributes to comprehensive, biopsychosocial treatment approaches.
